# Psoriasis and Major Adverse Cardiovascular Events: A Systematic Review and Meta‐Analysis of Observational Studies

**DOI:** 10.1161/JAHA.113.000062

**Published:** 2013-04-24

**Authors:** Ehrin J. Armstrong, Caitlin T. Harskamp, April W. Armstrong

**Affiliations:** 1 Division of Cardiovascular Medicine, University of California, DavisSacramento, CA (E.J.A.); 2 Department of Dermatology, University of California, DavisSacramento, CA (C.T.H., A.W.A.)

**Keywords:** cardiovascular diseases, epidemiology, meta‐analysis, myocardial infarction, psoriasis

## Abstract

**Background:**

Psoriasis is a chronic inflammatory disease that may be associated with increased risk of cardiovascular events, including cardiovascular mortality, myocardial infarction, and stroke.

**Methods and Results:**

We searched the MEDLINE, EMBASE, and Cochrane Central Register databases for relevant studies in English between January 1, 1980, and January 1, 2012. Extraction was by 3 independent reviewers. Summary incidence, risk ratios (RRs), and confidence intervals (CIs) were calculated using fixed‐effects and random‐effects modeling. Meta‐regression was also performed to identify sources of between‐study variation. Nine studies were included, representing a total of 201 239 patients with mild and 17 415 patients with severe psoriasis. The level of covariate adjustment varied among studies, leading to the possibility of residual confounding. Using the available adjusted effect sizes, mild psoriasis remained associated with a significantly increased risk of myocardial infarction (RR, 1.29; 95% CI, 1.02 to 1.63) and stroke (RR, 1.12; 95% CI, 1.08 to 1.16). Severe psoriasis was associated with a significantly increased risk of cardiovascular mortality (RR, 1.39; 95% CI, 1.11 to 1.74), myocardial infarction (RR, 1.70; 95% CI, 1.32 to 2.18), and stroke (RR, 1.56 95% CI, 1.32 to 1.84). Based on these risk ratios and the background population event rates, psoriasis is associated with an estimated excess of 11 500 (95% CI, 1169 to 24 407) major adverse cardiovascular events each year.

**Conclusions:**

Mild and severe psoriasis are associated with an increased risk of myocardial infarction and stroke. Severe psoriasis is also associated with an increased risk of cardiovascular mortality. Future studies should include more complete covariate adjustment and characterization of psoriasis severity.

## Introduction

Psoriasis is a chronic inflammatory disease of the skin and joints that affects 2% to 3% of the world's population.^1–2^ Recent research has emphasized that psoriasis is a systemic disease with multiple associated comorbidities.^3^ For example, patients with psoriasis also have an increased prevalence of cardiovascular risk factors including hypertension, diabetes, obesity, and dyslipidemia.^4–7^ These findings have led to the recommendation that all patients with psoriasis should undergo detailed screening and management of cardiovascular risk factors.^8^

Patients with psoriasis may also have an increased risk of major adverse cardiovascular events (MACE) beyond that attributable to measured cardiovascular risk factors.^9^ In support of this theory, large epidemiologic studies have found increased rates of cardiovascular mortality, myocardial infarction (MI), and stroke among patients with both mild and severe psoriasis.^10–12^ Shared inflammatory pathways, including TH1‐mediated inflammation, alterations in angiogenesis, and endothelial dysfunction, may link the pathogenesis of psoriasis with the development of atherosclerosis and cardiovascular disease.^13–14^ However, the magnitude of this association remains controversial, and it is uncertain whether the increased risk for MACE is limited only to patients with severe psoriasis.

To answer these questions, we performed a systematic review and meta‐analysis of the association between psoriasis and cardiovascular death, MI, and stroke. We stratified our analysis by mild versus severe psoriasis and included adjusted risk estimates accounting for comorbidities. Based on these results, we also estimated the attributable risk of psoriasis to excess major adverse cardiovascular events in the US population.

## Methods

### Selection of Studies

We systematically searched the MEDLINE, EMBASE, and Cochrane Central Register databases with the following search terms: “Psoriasis”[Mesh] AND {(“Death, Sudden, Cardiac”[Mesh]) OR (“Myocardial Infarction”[Mesh]) OR (“Stroke”[Mesh]) OR (“Cardiovascular Diseases”[Mesh])}. Our search was limited to English‐language and human‐only studies published between January 1, 1980, and January 1, 2012. The search yielded 558 results. All abstracts were read to determine eligibility for inclusion in the systematic review. To be included, original studies needed to fulfill the following inclusion criteria: case–control, cross‐sectional, cohort, or nested case–control design; evaluation of MI, stroke, cardiovascular death, or composite cardiovascular end point in conjunction with psoriasis; and analyses that compared psoriasis patients with control groups. The studies had to evaluate the incidence of subsequent cardiovascular death, MI, or stroke, with these 3 entities defined as overall MACE. The end point could be identified by physical examination, patient self‐report, medical chart review, or medical billing codes. A number of studies assessed MI or stroke prevalence but not incidence. These studies are detailed in Tables S1 and S2 but were not included in the analysis because they did not assess incidence.

### Data Extraction and Clinical Endpoints

The Meta‐Analysis of Observational Studies in Epidemiology (MOOSE) guidelines were used to guide analysis.^15^ The systematic review and data extraction were performed independently by 3 reviewers (E.J.A., C.T.H., and A.W.A.), and any differences were adjudicated by consensus. For each study included, we recorded the study year, country in which the study population lived, setting in which the study took place, study design, numbers of case and control subjects, age, sex, statistical adjustments for comorbidities, data collection processes (prospective versus retrospective), whether the results were a primary or secondary analysis of the publication, and whether psoriasis disease severity was assessed. A previously validated 6‐point scale was used to determine study quality, with values of 0 or 1 assigned to study design, assessment of exposure (psoriasis), assessment of outcome (major adverse cardiovascular events), control for confounding, evidence of bias, and assessment of psoriasis severity. Studies with a score of 0 to 3 were categorized as lower quality, whereas studies with scores of 4 to 6 were categorized as higher quality.^16^ Most of the included studies were of either case–control or cohort design. One study assessed the combined outcome of MACE.^9^ All others assessed MI, stroke, or cardiovascular death independently.

### Statistical Analysis

Because prior studies have suggested a significant effect modification of psoriasis severity on cardiovascular outcomes, we stratified our analysis on the basis of patients with mild psoriasis versus patients with severe psoriasis. To estimate the pooled risk ratio (RR), the adjusted effect size and reported upper and lower bounds of the 95% confidence interval for each study were log‐transformed. The inverse variance method was then applied with fixed‐effects and random‐effects models of DerSimonian and Laird.^17^ Study heterogeneity was assessed using the *I*^2^ statistic.

Risk ratios were used to calculate the excess risk for cardiovascular mortality, MI, and stroke among patients with psoriasis. Because 2 studies used standardized mortality ratios based on a population sample, we assumed that the control groups in each case consisted of an equal number of patients matched by age and sex with the same duration of follow‐up as the psoriasis group.^18–19^ In cases in which the total number of patient‐years of follow‐up was not reported, we integrated the mean of the aggregate data.^18^ In another study, the total patient‐years of follow‐up were available, but the total number of events was not reported.^20^ We therefore estimated the number of events on the basis of the size of the cohort and the reported events/1000 patient‐years.

Publication bias was assessed using visual inspection of a funnel plot of study size versus standard error, with formal statistical testing using the Begg adjusted rank correlation test.^21–22^ To explore sources of study heterogeneity, we performed meta‐regression using prespecified variables and fixed‐effects meta‐analysis. Prespecified sources of heterogeneity included study country, subject location (ambulatory or inpatient), multivariate adjustment for confounders, prospective versus retrospective study design, primary versus secondary analysis, ascertainment of psoriasis disease severity, measure of outcome, and study quality (0 to 3 versus 4 to 6).

To calculate the population attributable risk of psoriasis on major adverse cardiovascular events, we used the most current statistics from the American Heart Association,^23^ which are based on 2008 US census data.^24^ We assumed that a total of 7.5 million people in the United States have psoriasis, and that 10% of patients with psoriasis have severe psoriasis.^25^

All analyses were performed using STATA Version 11.2 (STATA Corp, College Station, TX). All statistical tests were 2 sided, with a significance level of <0.05.

## Results

### Study Selection

**Figure 1.**
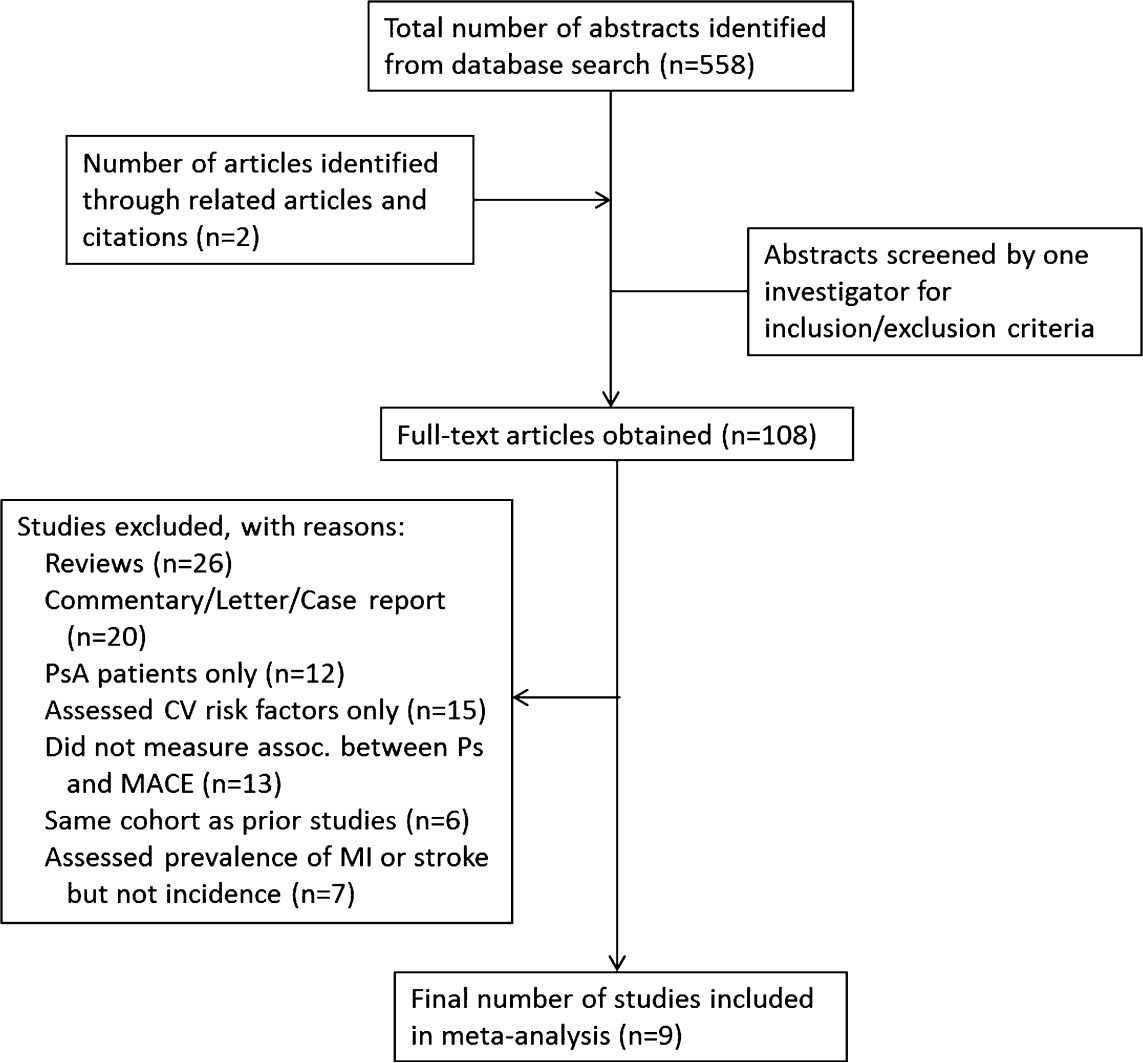
Article selection. CV indicates cardiovascular; MACE, major adverse cardiovascular events.

From the initial 558 search results, 108 full‐text articles were chosen for further review. Among these full‐text articles, 26 studies were excluded because they were reviews; 20 were letters, commentaries, or case reports; 12 exclusively assessed psoriatic arthritis (PsA) patients; 15 assessed cardiovascular risk factors only; 13 did not measure the association between psoriasis and MACE; 6 were of the same cohort as prior studies; and 7 assessed prevalence of MI or stroke but not incidence (Tables S1 and S2).^26–32^ Nine studies were therefore included in the meta‐analysis (Figure 1).^11–12,11–20,11–36^ Studies with significant cohort overlap (eg, in which multiple studies used the General Practice Research Database [GPRD] in overlapping periods) were included only once.^9–10,9–39^ In each case, the study with the highest‐quality measure and most complete reporting was included.

The baseline characteristics of each study, stratified by mild versus severe psoriasis, are shown in Tables 1 and 2. Two studies used standardized mortality ratios based on the expected mortality among patients matched for age and sex,^18–19,37^ whereas other studies used hazard ratios or rate ratios. Study designs included nested case–control, isolated cohorts based on practice patterns, or whole‐country cohort design. All studies except 1 differentiated mild from severe psoriasis, as defined by either inpatient status, need for phototherapy, or use of systemic medications.^34^

**Table 1.** Mild Psoriasis and Major Adverse Cardiovascular Events

Reference	Study Country	Number of Patients	Mean Age, Years	Events	Event Rate, Control	Event Rate, Psoriasis	Mean Follow‐Up Time, Years	Effect Measure	Definition of Outcomes	Adjusted Effect Size	Adjustment Variables
Cardiovascular mortality											
Mallbris et al^18^	Sweden	19 757	NR	1302	6.1	5.7	11.6	SMR	Death registry; ICD‐7, ICD‐8, and ICD‐9 codes	0.94 (0.89 to 0.99)	A, G
Ahlehoff et al^20^	Denmark	34 371	47.2	393	2.0	2.3	5.0	RR	Cardiovascular death using ICD‐10 code	1.14 (1.06 to 1.22)	A, G, M
Myocardial infarction											
Gelfand et al^11^	United Kingdom	127 129	46.4	2319	3.6	4.0	3.8	HR	Diagnostic code using READ or OXMIS	1.54 (1.24 to 1.91)	H, D, C, A, G, S, MI, BMI
Wakkee et al^34^*	Netherlands	15 820	48.9	223	2.3	2.3	6.0	HR	Hospitalization for MI	0.94 (0.8 to 1.11)	H, D, C, A, G, U
Ahlehoff et al^20^	Denmark	34 371	47.2	494	2.4	2.9	5.0	RR	MI using ICD‐10 code	1.22 (1.12 to 1.33)	A, G, M
Lin et al^35^	Taiwan	4162	NR	17	0.4	0.7	5.0	HR	New MI, using insurance database	2.10 (1.27 to 3.43)	H, D, C, A, G, SD
Stroke											
Gelfand et al^12^	United Kingdom	129 143	45.1	2100	4.0	3.7	3.7	HR	Diagnostic code using Read or OXMIS	1.06 (1.01 to 1.11)	A, G, H, D, C, S, N
Ahlehoff et al^36^	Denmark	36 765	46.1	838	3.1	4.5	5.0	RR	Ischemic stroke using ICD‐9 codes	1.25 (1.17 to 1.34)	A, G, SD, M

Event rates are reported as events/1000 person‐years. NR indicates not reported; SMR, standardized mortality ratio; ICD, International Classification of Diseases; RR, risk ratio; HR, hazard ratio; A, age; G, gender; M, medical comorbidities (individual comorbidities not reported); OXMIS, Oxford Medical Information System; H, hypertension; D, diabetes; C, cholesterol; S, smoking; U, healthcare utilization; N, neurovascular disease; MI, prior myocardial infarction; BMI, body mass index; SD, social demographics.

* Authors did not distinguish mild from severe psoriasis.

**Table 2.** Severe Psoriasis and Major Adverse Cardiovascular Events

Reference	Study Country	Number of Patients	Mean Age, Years	Events	Event Rate, Control	Event Rate, Psoriasis	Mean Follow‐Up Time, Years	Effect Measure	Definition of Outcomes	Adjusted Effect Size	Adjustment Variables
Cardiovascular mortality											
Mallbris et al^18^	Sweden	8991	NR	1529	10.6	16.2	10.5	SMR	Death registry; ICD‐7, ICD‐8, and ICD‐9 codes	1.52 (1.44 to 1.60)	A, G
Abuabara et al^33^	United Kingdom	3603	52.2	108	6.2	8.7	2.7	HR	Diagnostic code using READ or OXMIS	1.57 (1.26 to 1.96)	A, G
Ahlehoff et al^20^	Denmark	2621	46.9	41	2.0	3.1	5.0	RR	Cardiovascular death using ICD‐10 code	1.57 (1.27 to 1.94)	A, G, M
Stern et al^19^	USA	1376	46	246	7.8	8.0	22.4	SMR	Telephone interviews and national death index	1.02 (0.90 to 1.16)	A, G
Myocardial infarction											
Gelfand et al^11^	United Kingdom	3837	49.8	112	3.6	5.1	5.4	HR	Diagnostic code using READ or OXMIS	7.08 (3.06 to 16.36)	H, D, C, A, G, S, MI, BMI
Ahlehoff et al^20^	Denmark	2621	46.9	45	2.4	3.4	5.0	RR	MI using ICD‐10 code	1.45 (1.10 to 1.90)	A, G, M
Lin et al^35^	Taiwan	590	NR	5	0.4	1.7	5.5	HR	New MI, using insurance database	1.81 (0.69 to 4.74)	H, D, C, A, G, SD
Stroke											
Gelfand, 2009^12^	United Kingdom	3603	52.2	74	4.4	6.1	2.7	HR	Diagnostic code using READ or OXMIS	1.43 (1.10 to 1.87)	A, G, H, D, C, S, N
Ahlehoff et al^36^	Denmark	2793	46.0	90	3.1	6.8	4.7	RR	Ischemic stroke using ICD‐9 codes	1.65 (1.33 to 2.05)	A, G, SD, M

NR indicates not reported; SMR, standardized mortality ratio; ICD, International Classification of Diseases; RR, risk ratio; HR, hazard ratio; A, age; G, gender; M, medical comorbidities (individual comorbidities not reported); H, hypertension; D, diabetes; C, cholesterol; S, smoking; U, healthcare utilization; MI, prior myocardial infarction; BMI, body mass index; SD, social demographics; N, neurovascular disease, including prior stroke or transient ischemic attack.

### Quality of the Studies and Publication Bias

All studies were observational and included sufficient follow‐up to determine the end point of interest. All studies were deemed high quality (score of 4 or greater) using a prespecified 6‐point quality scale. Variable levels of covariate adjustment were performed (Tables 1 and 2), with all studies adjusting for age and sex, but only some studies including full adjustment for other medical comorbidities. The studies of cardiovascular mortality adjusted only for age, sex, and some medical comorbidities, whereas studies of myocardial infarction and stroke in general included more complete covariate adjustment. No evidence of publication bias was detected for cardiovascular mortality (*P*=0.7), MI (*P*=0.5), or stroke (*P*=0.9) using visual inspection of a funnel plot and formal testing with the Egger test.

Because observational studies may also have significant between‐study heterogeneity in design and cohort selection, we also performed meta‐regression analysis for the end points of cardiovascular mortality and MI (CV death in mild psoriasis and stroke were not included in meta‐regression testing because of identification of only 2 studies for each of these analysis subgroups and no significant between‐study heterogeneity). There was an association between study country and the strength of association of severe psoriasis with cardiovascular mortality (*P*=0.01), largely because the 1 US‐based study of cardiovascular mortality had a smaller reported RR than the other, European‐based studies.^19^ All other prespecified meta‐regression analyses were not statistically significant (Tables S3 through S5).

### Cardiovascular Mortality

**Figure 2.**
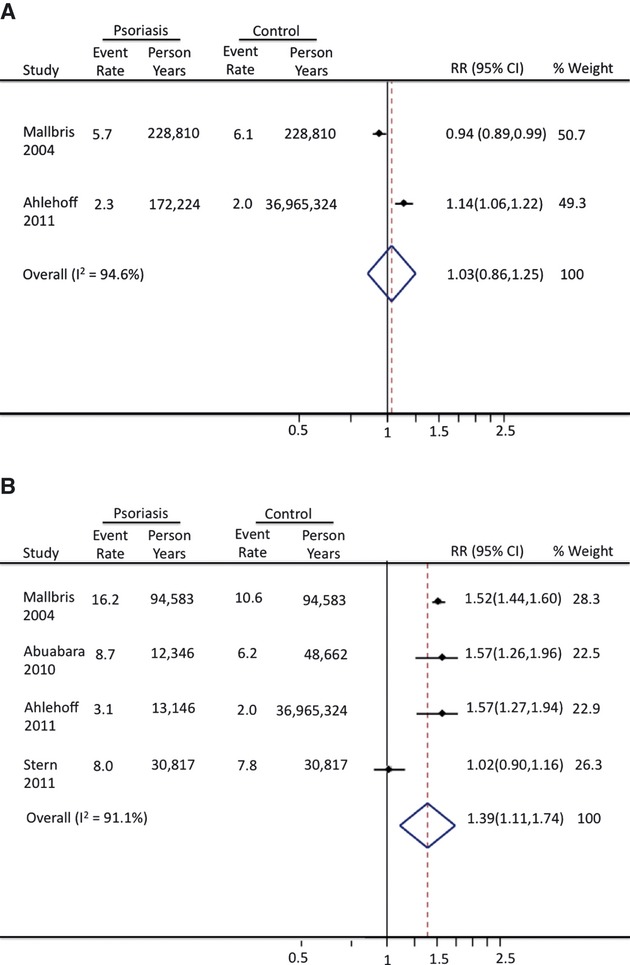
Cardiovascular death among patients with psoriasis. A, Risk of cardiovascular death among patients with mild psoriasis. B, Risk of cardiovascular death among patients with severe psoriasis. Rates are reported as events/1000 person‐years.

Cardiovascular mortality was studied among 4 cohorts, including patients from the United States, United Kingdom, Sweden, and Denmark (Figure 2). A total of 54 128 patients with mild psoriasis were studied. Only 2 studies addressed cardiovascular mortality among patients with mild psoriasis. The 2 studies had discordant findings, leading to no statistically significant association (RR, 1.03; 95% CI, 0.86 to 1.25) on meta‐analysis.

Among 16 591 patients with severe psoriasis, there was a significantly increased risk of cardiovascular mortality during long‐term follow‐up ranging from 2.7 to 22.4 years (RR, 1.39; 95% CI, 1.11 to 1.74). Discordant outcomes between the European‐based and US‐based studies accounted for all the between‐study heterogeneity (*I*^2^=91.1% before exclusion, *I*^2^=0 after exclusion). If the meta‐analysis was restricted to the 3 European‐based studies, the RR for cardiovascular mortality among patients with severe psoriasis increased to 1.53 (95% CI, 1.45 to 1.60). The incidence rate per 1000 person‐years for cardiovascular mortality among patients with severe psoriasis ranged from 3.1 to 16.2 (Table 2).

### Myocardial Infarction

**Figure 3.**
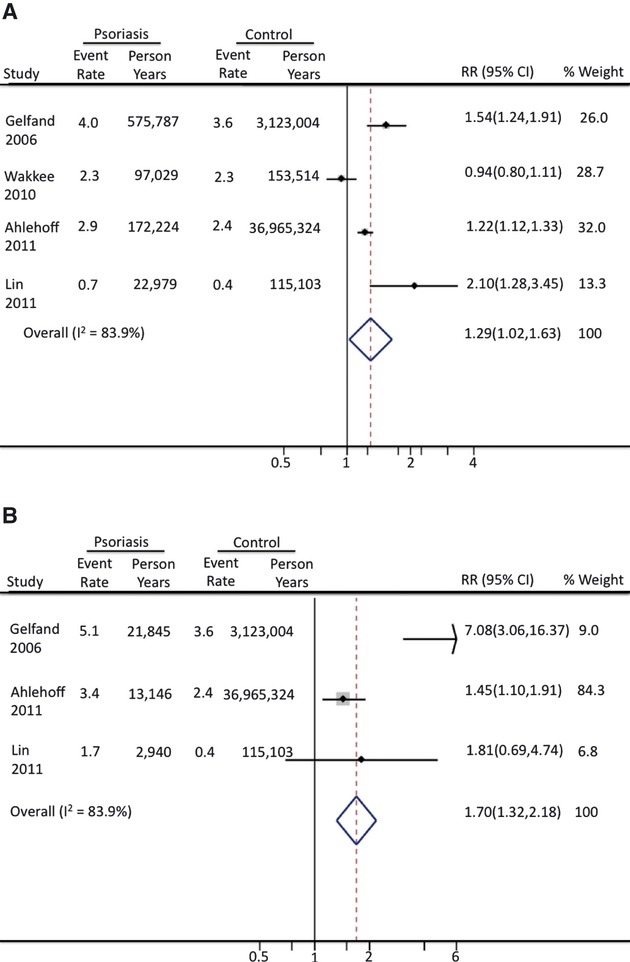
Myocardial infarction among patients with psoriasis. A, Risk of myocardial infarction among patients with mild psoriasis. B, Risk of myocardial infarction among patients with severe psoriasis. Rates are reported as events/1000 person‐years.

Myocardial infarction was studied among 4 cohorts (Figure 3). There was a significantly increased risk of MI among patients with both mild and severe psoriasis. Among the 181 492 patients with mild psoriasis, the RR of MI was 1.29 (95% CI, 1.02 to 1.63). For the 7048 patients with severe psoriasis, the RR of MI was 1.70 (95% CI, 1.32 to 2.18). In 1 study, patients with severe psoriasis were identified only by use of TNF‐alpha inhibitors.^35^ Excluding this study from the meta‐analysis did not significantly affect the outcomes (RR, 1.69; 95% CI, 1.30 to 2.19 for severe psoriasis). These studies were based on a total number of 3053 MI events among patients with mild psoriasis and of 162 MI events among patients with severe psoriasis. The incidence rate per 1000 person‐years for MI among patients with psoriasis ranged from 1.7 in a study conducted in Taiwan to 4.0 in a study conducted in the United Kingdom.^11,35^

### Stroke

**Figure 4.**
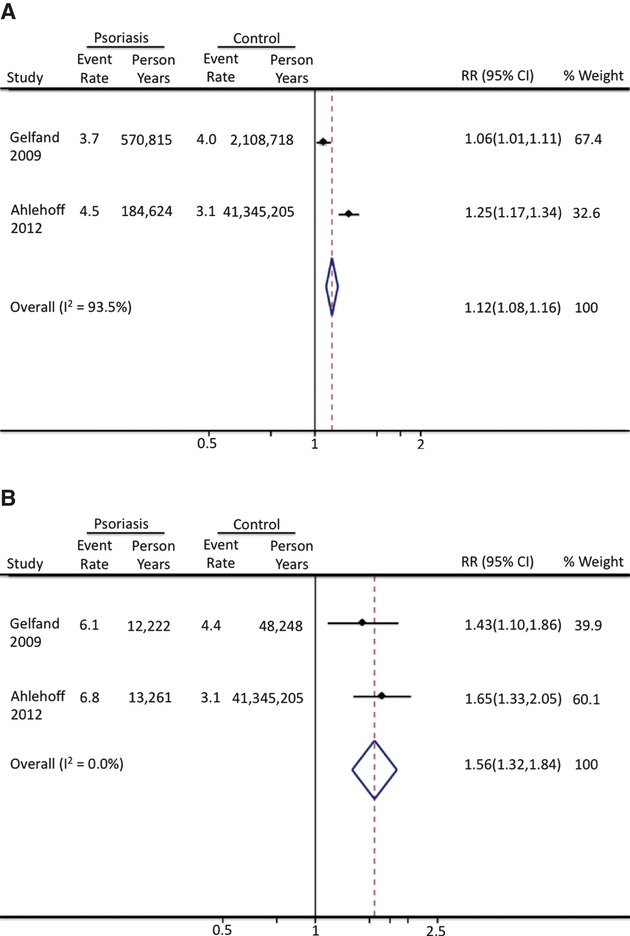
Stroke among patients with psoriasis. A, Risk of stroke among patients with mild psoriasis. B, Risk of stroke among patients with severe psoriasis. Rates are reported as events/1000 person‐years.

Two studies assessed the risk of incident stroke among patients with psoriasis (Figure 4). Among 165 908 patients with mild psoriasis, the RR for stroke was 1.12 (95% CI, 1.08 to 1.16). Among 6396 patients with severe psoriasis, the RR for stroke was 1.56 (95% CI, 1.32 to 1.84). Both these studies were derived from large European‐based cohorts and use of medical codes. In 1 study, patients with psoriasis were identified on the basis of medical prescriptions, and the analysis only included treated patients.^36^ The incidence rate per 1000 person‐years for stroke ranged from 3.7 to 5.0 for patients with mild psoriasis and from 6.1 to 6.8 for patients with severe psoriasis.

### Attributable Risk Estimate of Psoriasis

Using the most current background rates of cardiovascular mortality, myocardial infarction, and stroke in the US population, we calculated the population attributable risk of psoriasis on major adverse cardiovascular events (Table 3). On the basis of these estimates and pooling results from patients with mild and severe psoriasis, psoriasis in the United States is associated with an estimated 1269 (95% CI, −2208 to 5741) excess deaths from cardiovascular causes, 6479 (95% CI, 979 to 13 409) excess MIs, and 3782 (95% CI, 2399 to 5258) excess strokes each year, for an estimated total of >11 500 (95% CI, 1169 to 24 407) excess major adverse cardiovascular events each year.

**Table 3.** Population Attributable Risk of Psoriasis on Major Adverse Cardiovascular Events

	Baseline Rate Per 100 000/Year	Rate Ratio, Psoriasis (95% CI)	Rate Per 100 000/Year, Psoriasis	Excess Rate Per 100 000/Year, Psoriasis	Number of Excess Cases/Year Attributable to Psoriasis
Mild psoriasis					
CV death	256	1.03 (0.86 to 1.25)	263.7 (220 to 320)	7.7 (−36 to 64)	520 (−2419 to 4320)
MI	261	1.29 (1.02 to 1.63)	336.7 (266 to 425)	75.7 (5 to 164)	5109 (352 to 11 099)
Stroke	307.5	1.12 (1.08 to 1.16)	344.4 (332 to 356)	36.9 (25 to 49)	2491 (1661 to 3321)
Severe psoriasis					
CV death	256	1.39 (1.11 to 1.74)	355.8 (284 to 445)	99.8 (28 to 189)	749 (211 to 1421)
MI	261	1.70 (1.32 to 2.18)	443.7 (345 to 569)	182.7 (84 to 308)	1370 (626 to 2310)
Stroke	307.5	1.56 (1.32 to 1.84)	479.7 (406 to 566)	172.2 (98 to 258)	1292 (738 to 1937)

Estimates are based on 2008 US census data and current national rates of cardiovascular death, myocardial infarction, and stroke. The 95% CI estimates for the rate ratio are based on meta‐analysis results. CV indicates cardiovascular; MI, myocardial infarction.

## Discussion

The association between psoriasis and cardiovascular disease has gained increased attention in the past 5 years. Although psoriasis was once thought to be a disease limited to the skin, there is increasing awareness that patients with psoriasis have a number of associated medical comorbidities. These comorbidities may significantly affect quality of life and also place patients with psoriasis at higher risk of subsequent medical problems. Although many of the initial studies examining psoriasis and comorbidities assessed only the prevalence of risk factors, a number of recent cohort studies have assessed incident cardiovascular events among patients with psoriasis. In this meta‐analysis, we systematically assessed the incidence of MACE among patients with psoriasis to better understand the magnitude of this association and the additional contribution of psoriasis to cardiovascular disease.

In our analysis, we found that both mild and severe psoriasis were associated with significantly increased risk of MI and stroke. In addition, severe psoriasis was associated with significantly increased cardiovascular mortality. The strength of the association for MI and stroke was greater for severe than for mild psoriasis, further supporting a possible dose–response relationship between disease severity and the excess risk of cardiovascular disease. On the basis of the pooled risk ratios for mild and severe psoriasis, we estimated that psoriasis accounts for an additional approximately 11 000 major adverse cardiovascular events/year in the United States. Although the relative risk of MACE is greater for patients with severe compared with mild psoriasis, the greater population prevalence of mild psoriasis actually translates into a greater population attributable risk of mild psoriasis for both MI and stroke. These findings emphasize that all patients with psoriasis, rather than only those with severe psoriasis, should be educated regarding an increased risk of cardiovascular disease.

Prior studies have suggested an age interaction between psoriasis and cardiovascular risk, with younger patients having a significantly higher relative risk for cardiovascular disease than older patients.^11^ These risk estimates may reflect the bimodal incidence of psoriasis, with a differential effect of early‐onset psoriasis on progression of atherosclerosis. Alternatively, the development of additional cardiovascular risk factors coincident with aging may eventually outweigh the additional risk of psoriasis to cardiovascular disease. However, we recently found that even among older patient cohorts, patients with psoriasis undergoing coronary angiography were more likely to have coronary artery disease.^40^ Although we could not adjust in this meta‐analysis for an age‐dependent effect of psoriasis on cardiovascular outcomes, these findings should be widely applicable to the cohorts studied, in which the mean age ranged from 45 to 52 years of age. This age group represents a common age at which intervention into cardiovascular risk factors can substantially modify future cardiovascular risk.

Currently, no specific treatments exist for modification of cardiovascular risk independent of standard risk factors. In the absence of specific treatments, recognition of modifiable risk factors remains paramount. Recent survey results suggest that most physicians are not aware of the association between psoriasis and cardiovascular disease and that patients with psoriasis are not adequately screened for medical comorbidities.^41–42^ Once these modifiable conditions are recognized, aggressive lifestyle modification and medical intervention may be warranted. Recognizing the additional contribution of psoriasis to cardiovascular disease may also result in reclassification of a number of patients from low‐ or medium risk based on Framingham risk scores to a higher‐risk category.^43^

It is possible that treatment of psoriasis with systemic medications may independently affect cardiovascular outcomes. Methotrexate, which is commonly prescribed in cases of moderate to severe psoriasis, may reduce the risk of cardiovascular events, although most of this evidence is observational and based on patients with rheumatoid arthritis.^44^ TNF‐alpha inhibitors are increasingly used in the management of patients with moderate to severe psoriasis. Randomized trials with short duration of follow‐up showed no effect of TNF‐alpha inhibitors on cardiovascular events.^45^ Recently published observational data suggests that TNF‐alpha inhibitors may be associated with reduced incidence of cardiovascular events among patients with psoriasis.^46^ In addition, treatment of psoriasis with TNF‐alpha inhibitors may reduce the incidence of diabetes, thereby reducing long‐term cardiovascular risk.^47^ Although there is some concern that more recent IL 12/23 inhibitors may increase cardiovascular mortality, a recent meta‐analysis failed to find any association between these agents and cardiovascular events.^45^ Further research will be necessary to better delineate the effect of these systemic medications on cardiovascular events.

This study should be interpreted in the context of its design. First, observational studies have inherent limitations, including unmeasured confounders and between‐study heterogeneity. The included studies, however, were all high quality and included effect sizes that were adjusted. Second, a potential major limitation of this analysis is the extent of covariate adjustment performed in each primary study. For example, studies of cardiovascular mortality did not adjust for important covariates, including smoking and diabetes, both of which are known to occur with greater prevalence among patients with psoriasis. It is therefore possible that the apparent independent effect of psoriasis on cardiovascular mortality is partly attributable to incomplete covariate adjustment. The studies of myocardial infarction and stroke utsed more complete covariate adjustment including smoking and diabetes status, but not all these studies adjusted for body mass index, and patients with psoriasis are known to have a higher prevalence of obesity when compared with the general population. These analyses emphasize that future epidemiologic studies should include a more thorough assessment of cardiovascular risk factors among well‐defined cohorts of patients with psoriasis. Third, the majority of the studies used billing codes and/or medication prescriptions to identify patients with psoriasis. The study population therefore represents patients with treated psoriasis and may not reflect the entire, often undertreated population of patients with psoriasis. Furthermore, the definition of severe psoriasis varied between studies. Most cohorts identified only 3% to 10% of patients as having severe psoriasis, whereas recent estimates based on percent body surface area involvement suggest that 15% to 20% of patients with psoriasis have a moderate to severe form of the disease.^25^ Whether such patients have an intermediate risk profile between that of patients with mild versus severe psoriasis is uncertain. Fourth, the studied cohorts range over the last 1 to 3 decades. A number of new therapies have been developed for psoriasis in the past decade, and it is possible that these therapies have altered the current epidemiology of cardiovascular disease among patients with psoriasis.

In conclusion, this meta‐analysis supports a significant association between psoriasis and incidence of major adverse cardiovascular events, with a significant population attributable risk of psoriasis. Patients with psoriasis should be educated regarding the increased risk of cardiovascular disease and aggressively treated for modifiable cardiovascular risk factors. Further research into the mechanisms linking psoriasis with cardiovascular disease is warranted and may provide insights into both pathogenesis and treatment.
